# Sensing the environment by bacterial plant pathogens: What do their numerous chemoreceptors recognize?

**DOI:** 10.1111/1751-7915.14368

**Published:** 2023-11-06

**Authors:** Miguel A. Matilla, Tino Krell

**Affiliations:** ^1^ Department of Biotechnology and Environmental Protection Estación Experimental del Zaidín, Consejo Superior de Investigaciones Científicas Granada Spain

## Abstract

Bacteria have evolved multiple sensing strategies to efficiently adapt to their natural hosts and environments. In the context of plant pathology, chemotaxis allows phytopathogenic bacteria to direct their movement towards hosts through the detection of a landscape of plant‐derived molecules, facilitating the initiation of the infective process. The importance of chemotaxis for the lifestyle of phytopathogens is also reflected in the fact that they have, on average, twice as many chemoreceptors as bacteria that do not interact with plants. Paradoxically, the knowledge about the function of plant pathogen chemoreceptors is scarce. Notably, many of these receptors seem to be specific to plant‐interacting bacteria, suggesting that they may recognize plant‐specific compounds. Here, we highlight the need to advance our knowledge of phytopathogen chemoreceptor function, which may serve as a base for the development of anti‐infective therapies for the control of phytopathogens.

## Graphical abstract

Number of chemoreceptor genes for the 10 most relevant plant pathogens.

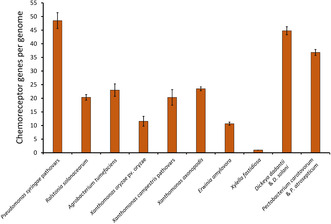

Chemotaxis permits bacteria to locate and move to places that are favourable for growth and survival. Typically, chemotactic signalling is initiated by the binding of chemoeffectors to ligand binding domains (LBD) of chemoreceptors, triggering a cascade that ultimately results in attraction or repellence responses. Most chemoeffectors are of metabolic value, suggesting that the access to nutrients is the major benefit of chemotaxis (Colin et al., 2021; Matilla et al., 2023). Alternatively, other chemoeffectors are rather niche‐specific signals like quorum sensing molecules (Zhang et al., 2020) or host‐derived factors such as mucin (Day et al., 2016), hormones (Antunez‐Lamas et al., 2009) and neurotransmitters (Pasupuleti et al., 2014) providing bacteria with important information on their environment and hosts.

## CHEMOTAXIS IS REQUIRED FOR VIRULENCE OF PATHOGENS OF VERY DIFFERENT LIFESTYLES

The capacity to perform chemotaxis was found to be essential for the virulence of pathogens with very different lifestyles, particularly during the early stages of the infective process (Matilla & Krell, 2018; Zhou et al., 2023). Chemotaxis represents thus a mechanism of major importance for the virulence of human and animal pathogens (Matilla & Krell, 2018), and the interference with the chemotactic mechanism in pathogens like *Helicobacter pylori* (Keilberg & Ottemann, 2016; Zhou et al., 2023), *Borrelia burgdorferi* (Motaleb et al., 2015) or *Campylobacter jejuni* (Korolik, 2019) significantly reduced their virulence. Notably, chemotaxis appears to be particularly important for plant pathogens since it is frequently required for efficient plant entry (Matilla & Krell, 2018). Plant openings like stomata and wounds secrete compounds that form gradients on plant surfaces. Bacteria sense these gradients and move to these openings facilitating plant entry, as a step prior to the subsequent infection of the plant host. Experiments with the global phytopathogen *Ralstonia solanacearum* illustrate well this issue. When tomato plants were spray‐inoculated with *R. solanacearum* or with non‐motile and non‐chemotactic mutants, a significant reduction in virulence was observed for the mutant strains, indicating that bacteria need to actively localize plant openings. In contrast, the virulence of wild‐type and mutant strains was comparable when bacteria were directly introduced into the plant (Yao & Allen, 2006). Another study showed that bacteria perform chemotaxis to open but not closed stomata (Melotto et al., 2006). Perturbing compound gradients on the leaf or root surface by excess chemoeffector was found to reduce the severity of virulence (Cerna‐Vargas et al., 2019; Tunchai et al., 2021). Taken together, the interference with chemotaxis is thus an alternative strategy to fight different pathogens (Matilla & Krell, 2023).

## SENSORY CAPACITIES OF CHEMORECEPTORS ARE ALMOST UNLIMITED

A large number of chemoreceptors in a wide range of bacteria have been characterized and their corresponding ligands identified (Matilla, Velando, Martín‐Mora, et al., 2022). The high number of chemoreceptors that respond to amino acids suggests that this may be the most relevant chemoeffector family (Matilla, Velando, Martín‐Mora, et al., 2022). Further ligands that were shown to interact with chemoreceptor LBDs include, for example, organic acids, sugars, polyamines, purines, nucleotides, quaternary amines or inorganic ions (Matilla, Velando, Martín‐Mora, et al., 2022). Without any doubt, this list will further grow in the future since new chemoeffectors are discovered regularly. In addition, some chemoreceptors mediate pH‐taxis (Tohidifar, Plutz, et al., 2020) or thermotaxis (Bi & Sourjik, 2018). This broad sensing capacity of chemoreceptors is also due to the variety of LBD types of different structures and sensing mechanisms (Sanchis‐López et al., 2021). Further features that broaden the sensing capacity of chemoreceptors are non‐canonical sensing mechanisms that do not involve the LBD [as exemplified by (Tohidifar, Bodhankar, et al., 2020) or receptor stimulation by the binding of ligand‐loaded solute binding proteins (Matilla et al., 2021).

## CHEMOSENSORY CAPACITIES REFLECT BACTERIAL LIFESTYLE

**FIGURE 1:**
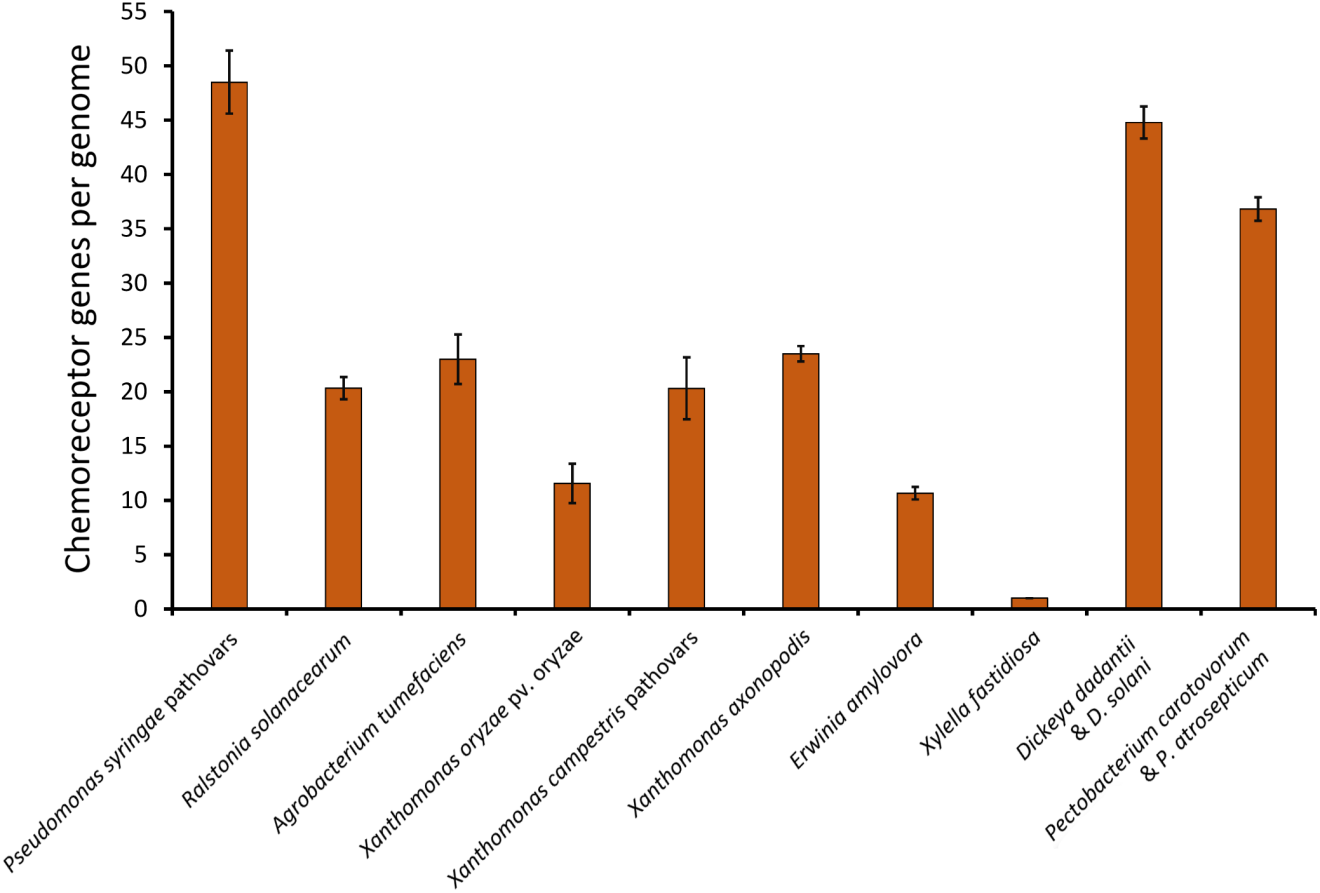
Number of chemoreceptor genes for strains of the top 10 most relevant plant pathogens as defined by Mansfield et al. (Mansfield et al., 2012). Only completed genomes were taken into account. Information extracted from the MIST 4.0 database (Gumerov et al., 2023).

The number of chemoreceptors in a particular bacterial species is an indication of the breadth of its chemosensory capacities. Initial studies have shown that the number of chemoreceptors varies significantly with the bacterial lifestyle (Lacal et al., 2010). For example, bacteria that inhabit a specific ecological niche have fewer chemoreceptors than bacteria that inhabit multiple and variable environments or that show a wide metabolic versatility. A more recent study derived from the analysis of almost 12,000 bacterial species revealed that almost half of their genomes contain chemoreceptor genes (Sanchis‐López et al., 2021). Notably, plant pathogens contain on average 27 chemoreceptors, whereas bacteria that do not interact with plants possess about 13 chemoreceptors (Sanchis‐López et al., 2021). This is best illustrated by the high number of chemoreceptors encoded by the 10 most relevant bacterial plant pathogens (Figure 1) (Mansfield et al., 2012). For example, *Pseudomonas syringae* strains, classified as the most relevant phytopathogenic bacteria (Mansfield et al., 2012), have about 50 chemoreceptor genes that represent about 1.4% of their genomes (Gumerov et al., 2023). Inspection of Figure 1 also shows that *Xylella fastidiosa* is the outlier since their genomes harbour a single chemoreceptor that is not involved in chemotaxis. Indeed, *X. fastidiosa* strains are non‐flagellated and their sole chemosensory signalling pathway regulates type IV pili‐based motility (Cursino et al., 2011). In contrast to the other phytopathogens listed in Figure 1, *X. fastidiosa* does not enter the host through plant surface openings, but is injected into the plant by xylem‐sap feeding insects (Landa et al., 2022). Therefore, the absence of a chemotactic system in *X. fastidiosa* supports the notion that chemotactic movements are required for the active entry of plant pathogens through surface openings.

## EVIDENCE FOR PHYTOPATHOGEN‐SPECIFIC CHEMORECEPTOR FAMILIES

The ligand specificity of a chemoreceptor is typically determined by its LBD. Sanchis‐López et al. (Sanchis‐López et al., 2021) retrieved more than 72,000 LBD sequences from chemoreceptors encoded in over 5,500 genomes and clustered these LBDs into thousands of families based on sequence similarities. For each of these clusters, the authors have then determined the relative abundance of plant‐associated bacterial species. They identified hundreds of clusters that are almost exclusively present in plant‐associated bacteria and species with a marked plant‐associated lifestyle like phytopathogens, suggesting that these receptors sense plant‐specific compounds. Certainly, the definition of plant‐specific chemoreceptor families is partially due to the phylogenetic proximity of different phytopathogenic strains (Sanchis‐López et al., 2021). However, the authors also demonstrate that many plant‐specific chemoreceptor families contain members from phylogenetically distant bacteria. LBDs are rapidly evolving domains (Gavira et al., 2020; Macadangdang et al., 2022) and evolutionary pressures resulting from plant interactions are likely to have generated many LBDs that sense plant compounds (Gavira et al., 2023).

## WHAT DO WE KNOW ABOUT PHYTOPATHOGEN CHEMORECEPTOR FUNCTION?

Considering the importance of chemotaxis in plant infection and the large number of phytopathogen chemoreceptors, our knowledge on their function is currently very scarce. This lack of knowledge is partly due to the fact that the major model species in chemotaxis research have alternative lifestyles. Research has so far shown that phytopathogen chemoreceptors respond to a number of compounds of the general metabolism like amino acids (Cerna‐Vargas et al., 2019; Kumar Verma et al., 2018; McKellar et al., 2015; Tumewu et al., 2021; Velando et al., 2023), organic acids (Hida et al., 2015, 2019), quaternary amines (Matilla, Velando, Tajuelo, et al., 2022) or nitrate (Gálvez‐Roldán et al., 2023; Monteagudo‐Cascales et al., 2023). Although these molecules are not plant‐specific compounds, they are present at high concentrations in plant exudates (Lohaus, 2022); Vives‐Peris et al., 2020) and are therefore major signals for host recognition. Notably, several mutant strains deficient in chemotaxis to amino acids, nitrate or malate showed reduced plant entry and/or virulence (Table 1). In contrast, only few chemoreceptors have been identified that respond to plant‐specific compounds. One example is the chemoattraction of *Dickeya dadantii* to the plant hormone jasmonate (Antunez‐Lamas et al., 2009). Two candidate jasmonate chemoreceptors have been identified, although the molecular mechanism of ligand perception remains unclear (Rio‐Alvarez et al., 2015). Mutants defective in chemoreceptors involved in jasmonate sensing showed reduced entry into *Arabidopsis thaliana* and mutant plants deficient in jasmonate biosynthesis were more resistant to *D. dadantii* invasion (Antunez‐Lamas et al., 2009; Rio‐Alvarez et al., 2015), supporting the notion that chemotaxis to this phytohormone is required for virulence (Antunez‐Lamas et al., 2009; Rio‐Alvarez et al., 2015). Chemotaxis to other phytohormones like auxins and salicylates was observed in various plant‐associated bacteria (Gavira et al., 2021; Rico‐Jiménez et al., 2022) and it appears likely that phytopathogens may also respond chemotactically to phytohormones other than jasmonate.

**TABLE 1:** Assessment of the effect of individual chemoreceptors on plant virulence.

Chemoreceptor^a^	Ligand	Species	Chemoreceptor mutant phenotype	Ref.
Chemoreceptor mutants with affected virulence				
PsPto‐PscA	Amino acids	*Pseudomonas syringae*	Reduced virulence in tomato	(Cerna‐Vargas et al., 2019)
PsPto‐PscC	GABA/L‐Pro	*P. syringae*	Reduced entry and virulence in tomato	(Santamaría‐Hernando et al., 2022)
PscB & PscC2	Amino acids	*P. syringae*	Reduced entry and virulence in tobacco	(Tumewu et al., 2021)
AerA & AerB	Aerotaxis	*P. syringae*	Reduced entry and virulence in tobacco	(Tumewu et al., 2022)
McpG	γ‐aminobutyric acid	*P. syringae*	Reduced entry and virulence in tobacco	(Tumewu et al., 2020)
Dd15070	Nitrate/nitrite	*Dickeya dadantii*	Reduced entry and virulence in potato	(Gálvez‐Roldán et al., 2023)
ABF‐0020167 & ABF‐0046680	Jasmonate/xylose	*D. dadantii*	Reduced entry into *Arabidopsis thaliana*. Jasmonate‐deficient plant mutants are more resistant to invasion	(Antunez‐Lamas et al., 2009; Rio‐Alvarez et al., 2015)
Mcp2	Amino acids	*Xanthomonas oryzae*	Reduced entry and virulence in rice	(Kumar Verma et al., 2018)
McpM	L‐malate	*Ralstonia pseudosolanacearum*	Reduced virulence in tomato	(Hida et al., 2015)
Aer2	Aerotaxis	*R. pseudosolanacearum*	Reduced virulence in tomato	(Yao & Allen, 2007)
Chemoreceptor mutants with virulence comparable to the wild‐type strain				
PscA	Amino acids	*P. syringae*	Unchanged entry and virulence in tobacco	(Tumewu et al., 2021)
	Amino acids	*R. pseudosolanacearum*	Unchanged virulence in tomato	(Hida et al., 2015)
McpT	D‐malate	*R. pseudosolanacearum*		(Tunchai et al., 2017)
McpB	Borate	*R. pseudosolanacearum*		(Hida et al., 2017)
McpC/McpP	Citrate	*R. pseudosolanacearum*		(Hida et al., 2019)

^a^ Only chemoreceptors with known chemoeffectors/signals are listed.

Our current knowledge suggests that chemotaxis chemosensory pathways are rather insulated circuits, indicating that ligand binding at chemoreceptor causes a single type of output, namely chemotaxis (Ortega et al., 2017). In this context, the observation of an apparent crosstalk of plant pathogen chemoreceptors with other signalling circuits is of interest. For example, a mutant in the PsPto‐PscA chemoreceptor in *P. syringae* showed reduced chemotaxis to several amino acids, but also altered c‐di‐GMP levels, biofilm formation and motility (Cerna‐Vargas et al., 2019). In analogy, a mutant in the γ‐aminobutyric acid (GABA)‐responsive chemoreceptor PsPto‐PscC of the same species showed altered expression of genes involved in GABA metabolism (Santamaría‐Hernando et al., 2022). Alternatively, a mutant strain of *D. dadantii* defective in the nitrate‐responsive chemoreceptor Dd15070 showed alterations in the expression of a number of genes involved in nitrate metabolism and virulence (Gálvez‐Roldán et al., 2023). In addition, a chemotactic deficient mutant in the response regulator CheY1 of *Xanthomonas oryzae* showed reduced biofilm formation and siderophore production ‐ phenotypes that were associated with changes in the expression of adhesin genes as well as in genes involved in iron uptake (Kumar Verma et al., 2018). Further research is therefore needed to elucidate the molecular mechanisms of the observed crosstalk and to establish to what degree this is a general phenomenon in phytopathogen chemoreceptor function.

## EXPERIMENTAL APPROACHES AIMED AT CLOSING THE GAP IN PHYTOPATHOGEN CHEMORECEPTOR FUNCTION

In the past, different in vivo and protein‐based in vitro approaches have been employed to identify chemoreceptor function. Each approach has its advantages and disadvantages. Our work has shown that although a chemoreceptor ligand had been identified by in vitro approaches, at times no or only marginal chemotaxis responses are observed by the wild‐type strain, indicating that under standard laboratory conditions, chemotaxis to the chemoeffector identified is not performed (Martín‐Mora et al., 2019). In this context, chemoreceptor expression in phytopathogens was shown to be tightly regulated, for example, during interaction with host plants (Kumar Verma et al., 2018; Yu et al., 2013) or under conditions that mimic those during plant‐bacteria interaction (Babujee et al., 2012; Hida et al., 2019; Santamaría‐Hernando et al., 2020)–an aspect that hampers the identification of novel chemoeffectors and chemoreceptors by analysing the chemotactic behaviour. In addition, the existence of multiple chemoreceptors with overlapping functions within the same bacterial strain may result in the absence of a chemotaxis phenotype for single receptor mutants due to the functional compensation by other chemoreceptors (Corral‐Lugo et al., 2018; Oku et al., 2017; Parales et al., 2013; Taha et al., 2022). To overcome these issues, a major in vitro approach consists of the purification of the individual chemoreceptor LBDs and to use them for high‐throughput ligand screening using compound libraries (Fernandez et al., 2018). Although this method identifies ligands that bind to LBDs, it fails to monitor chemoreceptor activation by non‐canonical mechanisms or stimulation by ligand‐loaded solute binding proteins. However, the combined use of in vivo and in vitro approaches is a powerful set‐up to identify the function of chemoreceptors in plant pathogens, as illustrated by a number of examples (Cerna‐Vargas et al., 2019; Gálvez‐Roldán et al., 2023; Monteagudo‐Cascales et al., 2023; Santamaría‐Hernando et al., 2022; Velando et al., 2023).

## KNOWLEDGE ON PHYTOPATHOGEN CHEMORECEPTOR FUNCTION AS BASE FOR THE DEVELOPMENT OF ANTIBACTERIAL STRATEGIES

The Food and Agriculture Organization (FAO) estimates that the agricultural production needs to increase its current productivity at least 70% by 2050 to meet the food needs of the world's growing population. This increase in production is being challenged by the impact of phytopathogens which, according to the FAO, are responsible for up to 40% of crop losses worldwide. Historically, phytopathogens are treated with chemical pesticides, but current regulations, e.g. those specified by the European Union Green Deal, are aimed at reducing the use of these pesticides (Roca & Matilla, 2023). In this regard, interfering with signalling pathways controlling virulence represents an innovative approach for the control of phytopathogens. For example, quorum quenching, or the interference with quorum sensing regulatory processes was shown to be a successful strategy for the control of various plant pathogens in different agricultural crops (Zhang et al., 2023). Among these novel strategies, interference with motility and chemotaxis is emerging as a promising anti‐infective therapy for the biocontrol of phytopathogens (Matilla & Krell, 2023). In this respect, it is crucial to advance our knowledge on the chemoeffectors that are recognized by chemoreceptors of phytopathogenic bacteria. Progress in this direction will allow, for example, the modulation of key virulence processes of phytopathogens such as chemotactic responses or biofilm formation.

## AUTHOR CONTRIBUTIONS

**Miguel A. Matilla:** Writing – original draft (equal); writing – review and editing (equal). **Tino Krell:** Writing – original draft (equal); writing – review and editing (equal).

## CONFLICT OF INTEREST STATEMENT

The authors declare no conflict of interest.
